# Draft Genome Sequence of *Campylobacter coli* Strain IPSID-1 Isolated from a Patient with Immunoproliferative Small Intestinal Disease

**DOI:** 10.1128/genomeA.00079-14

**Published:** 2014-03-13

**Authors:** Alexis Criscuolo, Arnaud de la Blanchardière, Solène Coeuret, Virginie Passet, Virginie Saguet-Rysanek, Michel Vergnaud, Renaud Verdon, Alexandre Leclercq, Marc Lecuit, Sylvain Brisse

**Affiliations:** a Institut Pasteur, Evolutionary Microbial Genomics Unit, Paris, France; b CNRS, UMR 3525, Paris, France; c Infectious Diseases Unit, University of Caen, Caen, France; d Department of Pathology, University of Caen, Caen, France; e Department of Microbiology, University Hospital of Caen, Caen, France; f Institut Pasteur, Biology of Infection Unit, Paris, France; g Institut National de la Sante et de la Recherche Medicale U1117, Paris, France; h Paris Descartes University, Sorbonne Paris Cité, Department of Infectious Diseases and Tropical Medicine, Necker-Pasteur Centre for Infectious Diseases, Necker-Enfants Malades University Hospital, Institut Imagine, APHP, Paris, France

## Abstract

The genome sequence and annotation of *Campylobacter coli* strain IPSID-1 are reported here. This bacterial isolate is the first to be cultured from a patient with immunoproliferative small intestinal disease (IPSID). The draft genome sequence is 1.683 Mb long, comprises 64 contigs, and has 31.26% G+C content.

## GENOME ANNOUNCEMENT

Gram-negative bacteria of the genus *Campylobacter* are often implicated in human and animal diseases, including enteritis, abortion, and septicemia. *Campylobacter jejuni* and *Campylobacter coli* are the medically most important species of the genus (1). Based on 16S rRNA gene sequencing, *in situ* hybridization, and immunohistochemistry, *Campylobacter* spp. have also been associated with immunoproliferative small intestinal disease (IPSID), a rare variant of B-cell mucosa-associated lymphoid tissue (MALT) lymphoma of the small intestine, characterized by the synthesis of a monotypic truncated immunoglobulin alpha heavy chain lacking an associated light chain (2, 3, 4). Recently, *C. coli* was isolated from the stool of a patient with ileocecal IPSID (5). Here, we report the draft genome sequence of this IPSID-associated *C. coli* strain, IPSID-1.

Whole-genome shotgun sequencing was performed using an Illumina HiSeq 2000 sequencer. Libraries were constructed using Nextera technology and sequenced using a 2 × 100 nucleotide paired-end strategy, leading to ~10,585,000 paired-read sequences. All reads were preprocessed to remove low-quality or artifactual bases. Library adapters, as well as base pairs occurring at 5′ and 3′ ends and supported by a Phred quality score <20, were trimmed off using AlienTrimmer (6). Reads of length <95 bp after the above cleaning steps were discarded, as well as those containing >20% bp with a Phred score of <20. Finally, the program fqduplicate (ftp://ftp.pasteur.fr/pub/gensoft/projects/fqtools) was used to discard every duplicate paired-end read. The remaining reads (~6,682,000 paired-end and ~2,176,000 single-end) were assembled using clc_assembler (version 4.10.86742) from the CLC Genomics Workbench analysis package (http://www.clcbio.com/products/clc-genomics-workbench/), with contig sequences of <500 nucleotides being discarded and with a de Bruijn graph *k*-mer parameter value of 57, which maximized the N_50_, N_75_, and N_90_ values (i.e., 302,476, 162,145, and 45,795 bp, respectively).

A total of 64 contigs organized into 38 scaffolds were obtained, with a total length of 1,683,384 bp. An average coverage depth of ~920× was obtained. The G+C content of the genome sequence is 31.26%. The sequences were submitted to the RAST server (7) for gene prediction and annotation, which led to 1,766 protein-coding sequences, 40 detected tRNA genes, and 3 rRNA regions. Extraction of gene sequences corresponding to the *Campylobacter* multilocus sequence typing scheme (8) showed that IPSID-1 belongs to sequence type 4956 (ST-4956), which is associated with clonal complex 828. The genome comprises genes homologous to the *cdtABC* cluster, coding for cytolethal distending toxin (9, 10).

The availability of the draft genome sequence of *C. coli* IPSID-1, the first *Campylobacter* isolate from a case of IPSID, will contribute to a better understanding of the pathophysiological mechanisms of this disease.

### Nucleotide sequence accession numbers.

This whole-genome shotgun project has been deposited at DDBJ/EMBL/GenBank under the accession no. CBXC000000000. The version described in this paper is the first version, CBXC01000000.
